# Cytoprotective Effect of Ferritin H in Renal Ischemia Reperfusion Injury

**DOI:** 10.1371/journal.pone.0138505

**Published:** 2015-09-17

**Authors:** Heather C. Hatcher, Lia Tesfay, Suzy V. Torti, Frank M. Torti

**Affiliations:** 1 Department of Cancer Biology, Wake Forest University School of Medicine, Winston-Salem, North Carolina, United States of America; 2 Department of Biochemistry, Wake Forest University School of Medicine, Winston-Salem, North Carolina, United States of America; 3 Comprehensive Cancer Center, Wake Forest University School of Medicine, Winston-Salem, North Carolina, United States of America; Lady Davis Institute for Medical Research/McGill University, CANADA

## Abstract

Oxidative stress is a major contributor to kidney injury following ischemia reperfusion. Ferritin, a highly conserved iron-binding protein, is a key protein in the maintenance of cellular iron homeostasis and protection from oxidative stress. Ferritin mitigates oxidant stress by sequestering iron and preventing its participation in reactions that generate reactive oxygen species. Ferritin is composed of two subunit types, ferritin H and ferritin L. Using an *in vivo* model that enables conditional tissue-specific doxycycline-inducible expression of ferritin H in the mouse kidney, we tested the hypothesis that an increased level of H-rich ferritin is renoprotective in ischemic acute renal failure. Prior to induction of ischemia, doxycycline increased ferritin H in the kidneys of the transgenic mice nearly 6.5-fold. Following reperfusion for 24 hours, induction of neutrophil gelatinous-associated lipocalin (NGAL, a urine marker of renal dysfunction) was reduced in the ferritin H overexpressers compared to controls. Histopathologic examination following ischemia reperfusion revealed that ferritin H overexpression increased intact nuclei in renal tubules, reduced the frequency of tubular profiles with luminal cast materials, and reduced activated caspase-3 in the kidney. In addition, generation of 4-hydroxy 2-nonenal protein adducts, a measurement of oxidant stress, was decreased in ischemia-reperfused kidneys of ferritin H overexpressers. These studies demonstrate that ferritin H can inhibit apoptotic cell death, enhance tubular epithelial viability, and preserve renal function by limiting oxidative stress following ischemia reperfusion injury.

## Introduction

Renal ischemia-reperfusion injury is thought to underlie the pathophysiology of a number of serious clinical conditions, including the abrupt reduction of kidney function (acute renal injury (AKI)) seen in patients with sepsis, renal transplantation, and other ischemic insults [1]. For these patients, mortality remains high, especially for patients admitted to the intensive care unit, where in-hospital mortality rates may exceed 50%. Patients who survive the acute episode remain at high risk of developing chronic kidney disease that may progress to end-stage renal disease [2].

The molecular mechanisms that underlie such injury are incompletely understood. Diminished oxygen and nutrient delivery, an accumulation of toxic products in the cells of the kidney, and the release of cytokines play important roles [3, 4]. In addition, the generation of reactive oxygen species (ROS) makes a critical contribution and is thought to underlie tubular epithelial cell damage and death by apoptosis and necrosis [5–8]. Accordingly, depletion of endogenous antioxidants and upregulation of the endogenous antioxidant enzyme heme oxygenase-1 (HO-1) accompanies ischemia reperfusion injury (IRI) [9], and drugs that ameliorate oxidative stress can attenuate IRI [10].

A major contributor to oxidative stress is elemental iron, particularly in its ferrous (Fe^+2^) form. Although iron is essential for normal cellular function and is a constituent of hemoproteins, iron-sulfur proteins, and other essential proteins in cellular metabolism [11, 12], excess free iron can participate in a number of reactions that generate free radicals, including the iron-catalyzed Haber-Weiss reaction (Fenton chemistry). This reaction results in the generation of highly reactive hydroxyl radicals that can directly damage DNA, lipids and proteins, leading to cellular damage or cell death [13]. Evidence points to a detrimental role for iron in multiple models of AKI. For example, chelation of iron elicited protection in both *in vitro* and *in vivo* models [7, 8, 14–16].

Ferritin is a protein with the ability to sequester iron in a nontoxic but bioavailable form and thus limit free radical generation triggered by inflammation and other insults [17, 18]. Numerous studies have suggested a role for ferritin in protection from oxidative stress [19–22]. Ferritin is composed of two subunits, termed H and L. Mammalian ferritin is found in most tissues, with the ratio of H-to-L subunits dependent on tissue type and physiologic status of the cell; moreover, ferritin subunit composition can be readily modified in response to inflammatory and infectious conditions, as well as other stimuli [11, 23, 24]. Ferritin H and L subunits are encoded by separate genes and have separable functions: only ferritin H possesses ferroxidase activity that oxidizes Fe (II) to Fe (III) as iron is internalized and sequestered in the ferritin mineral core; ferritin L is associated with iron nucleation and protein stabilization [17, 18, 25–27]. Ferritin regulation occurs at multiple levels [28, 29]. At the translational level, ferritin is primarily regulated by the binding of iron regulatory proteins (IRPs) to a stem-loop secondary structure termed the iron responsive element (IRE) in the 5’-untranslated region (5’-UTR) of H and L ferritin mRNA (reviewed in [24, 30, 31]. The proinflammatory cytokines tumor necrosis factor-α (TNFα) and interleukin-1α (IL-1α) also modulate ferritin subunit composition and content through transcriptional induction of the ferritin H subunit [32–34]. Increased ferritin expression has been reported in numerous pathophysiological settings, including atherosclerosis, cancer, and neurodegenerative diseases[24].

Few studies have assessed the ability of ferritin to ameliorate oxidative stress *in vivo*. In one study, overexpression of ferritin H in the dopaminergic SN (substantia nigra) neurons of the mouse brain reduced oxidative stress markers following the administration of a Parkinson’s-inducing agent 1-methyl-4-phenyl-1,2,3,6-tetra-pyridine [20]. Conversely, knockout of one allele of ferritin H increased markers of oxidative stress in mouse brains [35]. In another study, *ex vivo* delivery of recombinant adenovirus expressing the ferritin H gene was shown to have an anti-apoptotic function that protected rat livers from ischemia reperfusion injury and prevented hepatocellular damage upon transplantation into syngeneic recipients [19].

The role of ferritin H in another form of nephrotoxicity has been previously assessed using proximal tubule-specific ferritin H knockout mice [22]. In this study, ferritin H knockout mice exposed to cisplatin or glycerol-induced rhabodomyolysis demonstrated significant mortality, worse structural and functional renal injury, and increased levels of apoptosis when compared to controls [22]. Although these experiments demonstrated that a reduction in levels of ferritin H results in increased cytotoxicity, they did not test whether over-expression of ferritin H could in itself attenuate nephrotoxicity. The findings reported here not only demonstrate a direct protective effect of ferritin H overexpression on renal injury, but identify ischemia/reperfusion as a different and clinically important mode of renal injury in which ferritin is reno-protective.

To test whether ferritin H exerts a cytoprotective function in the kidney of mice exposed to renal ischemia/reperfusion injury, we took advantage of an *in vivo* model enabling conditional, doxycycline-regulated, tissue-specific expression of ferritin H in the mouse kidney established in our laboratory (52). The expression of the ferritin H transgene in this mouse is driven by the LAP promoter, which leads to prominent overexpression in the renal cortex as well as mildly increased expression in the liver [36]. Increasing renal ferritin H expression significantly alters iron metabolism in the kidneys of these transgenic mice, inducing a phenotype of iron depletion in the kidney [36]. We assessed the response of these conditional ferritin H transgenic mice to renal ischemia reperfusion injury. In particular, we assessed whether ferritin modulates oxidative injury, apoptosis, and acute tubular necrosis, key features of ischemia reperfusion injury [37, 38].

## Materials and Methods

### Ferritin H overexpressing mice

All procedures outlined in these studies were approved by the Wake Forest University Institutional Animal Care and Use Committee. Experiments were performed in FVB/N mice containing three integrated transgenes, as previously described [36]. In particular, these mice contain two genes driven by a doxycycline-dependent bicistronic promoter: ferritin H with a mutated iron responsive element (IRE) (to override translational regulation of ferritin H by iron), and enhanced green fluorescent protein (EGFP). In addition, they contain an optimized Tet-On transactivator (rtTA2S-S2LAP) to drive doxycycline-dependent expression of these genes. The presence of transgenes was confirmed using PCR as described [39]. These FerHmIRE/TetO7/EGFP ×rTA LAP-1 transgenic mice are referred to as “LAPFerH” mice in the present study. Our previous work has shown that doxycycline treatment of LAPFerH mice induces expression primarily in the kidney, and engenders a phenotype of renal iron depletion [36]. Both male and female animals aged 4–7 months were used. Because consistent and sustained expression of ferritin H was critical to our study and longer exposure to doxycycline would allow more cells to become induced, LAPFerH (experimental, n = 9) or rTA LAP-1 transactivator-only (control, n = 10) animals received a subcutaneous doxycycline time release pellet (42 mg) per manufacturer’s protocol (Innovative Research of America, Sarasota, FL) that released a dose of 0.7 mg/kg/day for 60 days followed by oral administration of doxycycline, 2 mg/mL in drinking water containing 2% sucrose, administered for an additional 10–14 days immediately prior to renal ischemia reperfusion injury. Doxycycline-containing sucrose water bottles were exchanged every 2–3 days. Mice were sacrificed on day 60 after implantation of the doxycycline pellet and 24 hours after ischemia/reperfusion injury (see below).

### Ischemia reperfusion injury model

Immediately prior to surgery, urine was obtained from mice by applying gentle pressure to the abdomen to induce urination onto a piece of Parafilm. The collected urine was placed in an Eppendorf tube and stored at -80°C until analysis. Mice were anesthetized by isoflurane inhalation (2–3% induction, 2% maintenance) and underwent bilateral flank incisions and dissection of the renal pedicles. A microvascular clamp was placed on the right renal pedicle for 45 min while the animal was kept at constant temperature (continuously monitored) and well hydrated. The left kidney served as the sham treatment without clamping. After 45-min ischemia, the clamp was removed, the wounds were sutured, and mice were allowed to recover. Immediately following surgery, the animal was removed from inhalant anesthetic and placed on a heating pad (lowest setting) for 10–15 min until alert and moving. The animal was then returned to its cage and observed for signs of distress every 15–30 min for the next 2 hrs before being returned to the vivarium. None of the animals displayed any adverse reactions and did not require additional veterinary care or analgesics after surgery. After 24 hrs reperfusion, the mice were euthanized by CO_2_ asphyxiation followed by exsanguination, urine was collected directly from the bladder using a 21g needle attached to a 3 mL syringe, and both kidneys were removed and placed in cold saline. EGFP expression was visualized at necropsy using a fiber optic epi-configured light source with 470/40 nm (excitation) filter set, and a 515 nm (emission) viewing plate (Lightools Research, Encinitas, CA) to confirm ferritin H overexpression in the kidneys of the LAPFerH animals. Kidneys were cut in half coronally, with one half fixed in 10% neutral buffered formalin for paraffin embedding and sectioning for histopathology and immunostaining. The remaining half was immediately frozen in liquid nitrogen and stored at -80°C until processed for protein analysis.

### Renal function monitoring

Neutrophil gelatinous-associated lipocalin, NGAL, is a protein that specifically reflects renal tubular damage and indicates renal dysfunction [40]. Immediately prior to surgery and at 24 hr reperfusion, urine was collected and frozen until time of measurement. Upon thawing in room temp water bath, urine samples were vortexed and centrifuged at 14,000 rpm x 5 min, and NGAL levels were measured using the Mouse ELISA kit (BioPorto Diagnostics #042-kit, Cedarlane Laboratories Ltd., Burlington, NC) per manufacturer’s instructions.

### Histological examination

Formalin-fixed paraffin embedded renal tissues were sectioned and stained with periodic acid–Schiff (PAS) reagents (#395B, Sigma-Aldrich Chemical, St. Louis, MO) and counterstained with hematoxylin. Tubular damage in PAS-stained sections was examined from a digital image captured at x200 magnification. The number of intact nuclei (blue) per 30 tubular profiles in the cortex and outer medulla per section were counted in blinded fashion; additionally, the proportion of tubular profiles (cortex and outer medulla) containing luminal cast material was measured by accessing digital images acquired at x200 magnification from three fields of view per section as previously described [41, 42].

### Assessment of apoptosis

To assess apoptosis, activated caspase-3 was evaluated using an antibody that detects cleaved caspase-3, a final step of the caspase activation cascade. Activated caspase-3 immunohistochemistry is recommended for the detection and quantification of apoptosis in tissue sections [43]. Immunohistochemistry was performed on deparaffinized tissue sections and subjected to heat-induced epitope retrieval by incubation in an aqueous solution of citraconic anhydride (0.05%, pH 7.4) for 45 min in a vegetable steamer, followed by 20-min cool-down. The sections were blocked in 2.5% horse serum in Tris buffer, pH 7.5 for 30 min at room temp in a humidified chamber, then incubated overnight at 4°C in a humidified chamber with rabbit polyclonal cleaved caspase-3 antibody (1:200 dilution) (#9661, Cell Signaling Technologies, Beverly, MA), followed by rabbit IgG secondary antibody conjugated to horseradish peroxidase and development using diaminobenzidine (DAB) (Vector Laboratories, Burlingame, CA, USA). The number of activated cleaved caspase-3– positive cells in the outer medulla and cortex were counted and expressed as the number of cells per three randomly selected fields of view for each mouse under x200 magnification.

### Western blotting analyses

Frozen tissue lysates were homogenized in ice cold lysis buffer [25 mM Tris, pH 7.4, 1% Triton x100, 1% sodium dodecyl sulfate, 1% sodium deoxycholate, 150 mM sodium chloride, 2 ug/mL aprotinin, 1 mM PMSF, complete EDTA-free protease inhibitor cocktail tablet (Roche Applied Science, Indianapolis, IN)], and total protein concentration was measured using a BCA protein assay kit (Pierce, Rockford, IL). Equal amounts of protein were separated on a 4–15% gradient sodium dodecyl sulfate-polyacrylamide gel (SDS-PAGE) and transferred to nitrocellulose membranes using a semi-dry blotting apparatus (Bio-Rad, Hercules, CA). Following Ponceau staining, each membrane was blocked in 5% powdered milk/TBS-0.1% Tween 20 (ferritin H) or 5% bovine serum albumin (4-HNE) for 2 hr with gentle shaking at room temperature prior to antibody applications. Briefly, immunoblotting antibodies and procedures used in this study included mouse monoclonal ferritin H (BioSource, Camarillo, CA); rabbit polyclonal β-actin (Abcam, Cambridge, MA); rabbit polyclonal GFP (Cell Signaling Technology) in 2.5% powdered milk/TBS-0.1% Tween 20, and mouse monoclonal 4-HNE (15 ug/mL, #MHN-20, GENOX, Baltimore, MD) in 5% bovine serum albumin/TBS-0.1% Tween 20 with gentle shaking overnight at 4°C followed by goat anti-mouse peroxidase-conjugated secondary antibody (1:5000; Jackson ImmunoResearch Laboratories, West Grove, PA) applied to each membrane in respective diluent for a minimum of 30 min at room temperature. Immunoreactive proteins were detected using an enhanced chemiluminescent reagent (#34080, SuperSignal West Pico Chemiluminescent Substrate, Thermo Scientific, Rockford, IL) according to the manufacturer’s instructions. The bands were visualized using the ImageQuant LAS 4000 digital imaging system (GE Healthcare, Piscataway, NJ). Adobe PhotoShop CS6 was used to perform denistometric analysis of protein levels and was normalized to β-actin protein levels. Where the number of samples was too large to include on a single gel, each gel was loaded with an equal number of control and experimental samples to facilitate comparisons. Images shown are representative of all animals in the group.

### Statistical analysis

All data were expressed as mean ± standard deviation. Differences were analyzed by one-way analysis of variance (ANOVA). Statistical calculations were performed using the SigmaPlot 12.0 (Systat Software Inc, San Jose, CA, USA). Significant difference was taken as P ≤ 0.05.

## Results

### Ferritin H preserves renal morphology and function following renal ischemia-reperfusion injury

**Fig 1:**
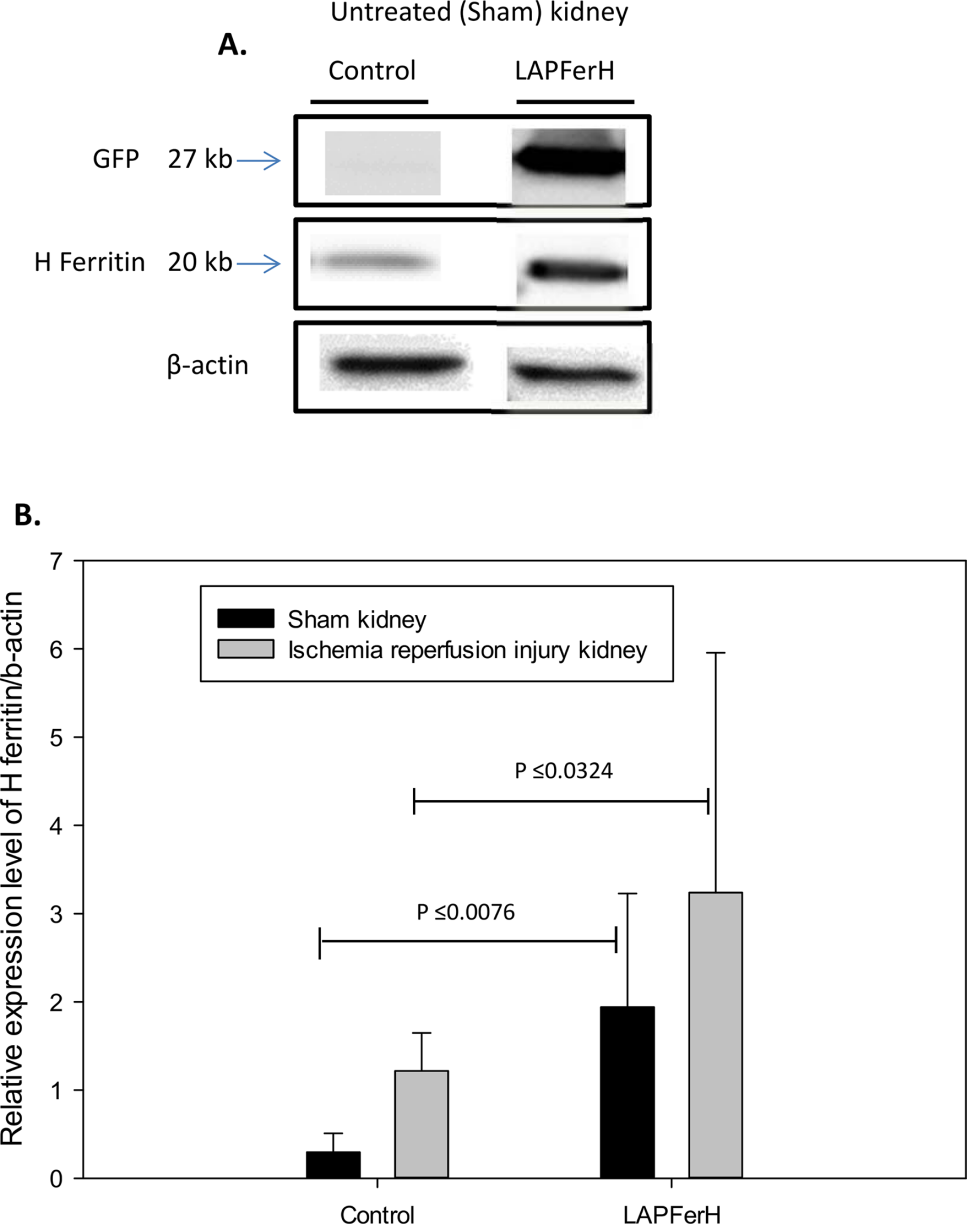
Expression level of ferritin H in kidneys pre- and post-ischemia-reperfusion injury. Following administration of doxycycline, ferritin H is overexpressed in the kidney of the LAPFerH mice (n = 9) compared to control mice (n = 10). (A.) Representative immunoreactive bands for GFP, ferritin H and β-actin are shown for untreated (sham) control and LAPFerH kidney protein lysates. (B.) Graph shows densitometry of relative expression of ferritin H /β-actin in untreated (sham) kidneys was 6.5-fold higher in the ferritin H overexpressing mice (LAPFerH) following administration of doxycycline compared to the control mice (P ≤ 0.0076, sham control vs. sham LAPFerH). Ischemia-reperfusion injury significantly increased ferritin H in LAPFerH mice vs. control (P ≤ 0.0324). All bands shown were obtained from the same gel and are representative of all animals in the group.

As expected and previously observed [36], following doxycycline treatment, green fluorescent protein (GFP) driven by the bicistronic promoter that also drives ferritin H was observed only in the kidneys of the conditional ferritin H-expressing mice (termed LAPFerH mice) and not in the control mice carrying only the LAP transactivator, indicating tight control of ferritin H expression by doxycycline (Fig 1A). Ischemia/reperfusion injury was induced by surgical clamping of the kidney for 45 min followed by removal of the clamp and 24 hr reperfusion (see Materials and Methods). The contralateral kidney was surgically exposed but not clamped, and served as the sham control. Ferritin H protein was induced more than 6.5-fold by doxycycline in the sham-treated kidneys of LAPFerH mice (n = 9) compared to the control mice (n = 10)(P ≤ 0.0076) (Fig 1A and 1B). Ischemia reperfusion injury increased ferritin H in both control and LAPFerH mice, although the increase was only statistically significant in the control mice (P ≤ 0.001)(Fig 1B). This increase in ferritin H in both control and LAPFerH mice was expected, given the previously observed response of ferritin to other nephrotoxic and stress-related insults [22]; however, ferritin levels were higher in LAPFerH mice compared to control mice both prior and subsequent to injury (P ≤ 0.0324) (Fig 1B). Use of this transgenic system thus increased both the extent and kinetics of ferritin H induction. Because doxycycline effectively induced ferritin H and the induction of ferritin H preceded ischemia reperfusion injury, it was possible to assess the cytoprotective effect of ferritin.

**Fig 2:**
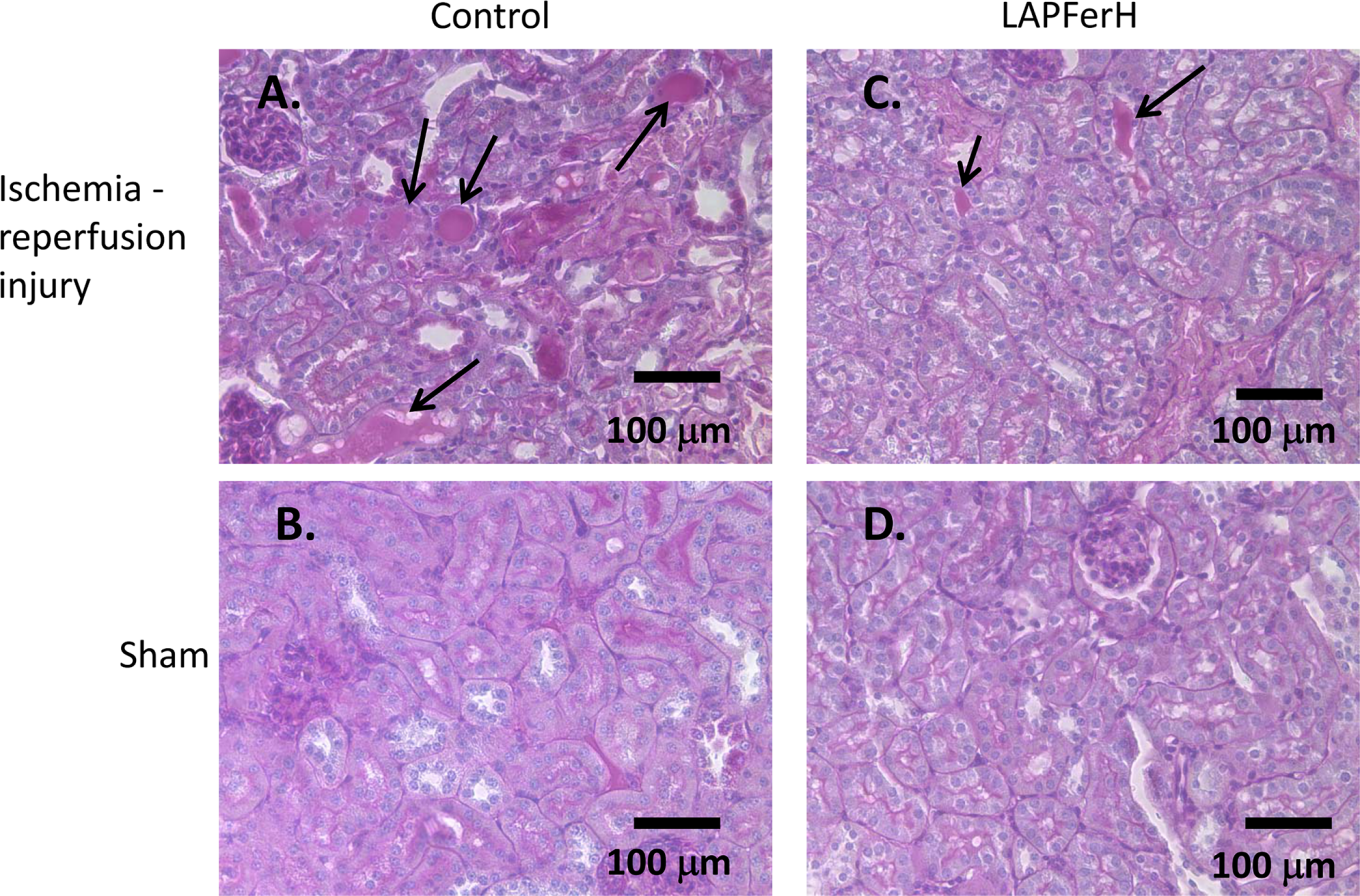
Histopathologic examination of kidney sections reveals changes consistent with ischemia-reperfusion injury. Mice were subjected to 45 min renal ischemia to the right kidney and then allowed to recover for 24 hr. The contralateral left kidney was sham treated. Periodic acid-Schiff (PAS) stained kidney sections are shown in panels A-D]. Note the degree of loss of brush border and tubular sloughing in the cortical kidney (arrows) following ischemia-reperfusion injury is greater in the Control (A.) compared to the LAPFerH (C.) mouse. Normal tubular morphology is observed in the sham kidneys of both Control (B.) and LAPFerH animals (D.)

Light microscopic analysis of PAS-hematoxylin-stained kidney sections revealed morphologic changes in both LAPFerH and control treatment groups typical of ischemic acute tubular injury[41]. Fig 2 shows that the brush border of proximal tubules in ischemia-reperfused kidney sections is thinned or absent and hyaline, granular, cellular and hemorrhagic casts are prominently stained with PAS in the lumen of many tubules (Fig 2A vs 2C) compared to the sham kidney sections (Fig 2B vs 2D). The degree of renal damage was markedly reduced in kidneys from mice overexpressing ferritin H. The number of luminal casts was 40.1% less in the ischemia-reperfused kidney sections of the LAPFerH mice compared to the control mice (16.0 ± 4.0, n = 9 vs. 24.0 ± 8.3, n = 10, respectively, per total number of tubule profiles from 3 fields of view, P ≤ 0.017)(Table 1).

**Table 1:** Histological evaluation of PAS-stained mouse cortical kidney sections.

Group	# intact nuclei/10 tubule cx				# luminal areas with cast		
	Sham	P value	IRI	P value	Sham	IRI	P value
**Control**	61.3 ± 12.7	n/s	41.6 ± 12.5	P≤0.0024	n/a	24.0 ± 8.3	P≤0.0170
**LAPFerH**	59.5 ± 14.6		58.3± 6.8		n/a	16.0± 4.0	

Values are mean ± SD.

The number of intact nuclei provides evidence of viable tubular epithelial cells that have survived following ischemia reperfusion injury, which has been shown to correlate with early regeneration of tubular cells [44–46]. Although our experiments were not designed to distinguish between protection against tubular damage and tubular regeneration, ischemia-reperfused kidney sections of the LAPFerH mice demonstrated a significantly increased number of intact nuclei compared to the ischemia-reperfused kidney sections of the control animals (58.3 ± 6.8 vs. 41.6 ± 12.5 per 30 tubular profiles, respectively, P ≤ 0.0024)(Table 1). The average number of intact nuclei in the sham kidney sections of all animals was 60.5 ± 13.3 per 30 tubular profiles per slide; thus, overexpression of ferritin H preserved 98% of nuclei in the ischemia-reperfused kidney sections compared to only 69% in control sections.

**Fig 3:**
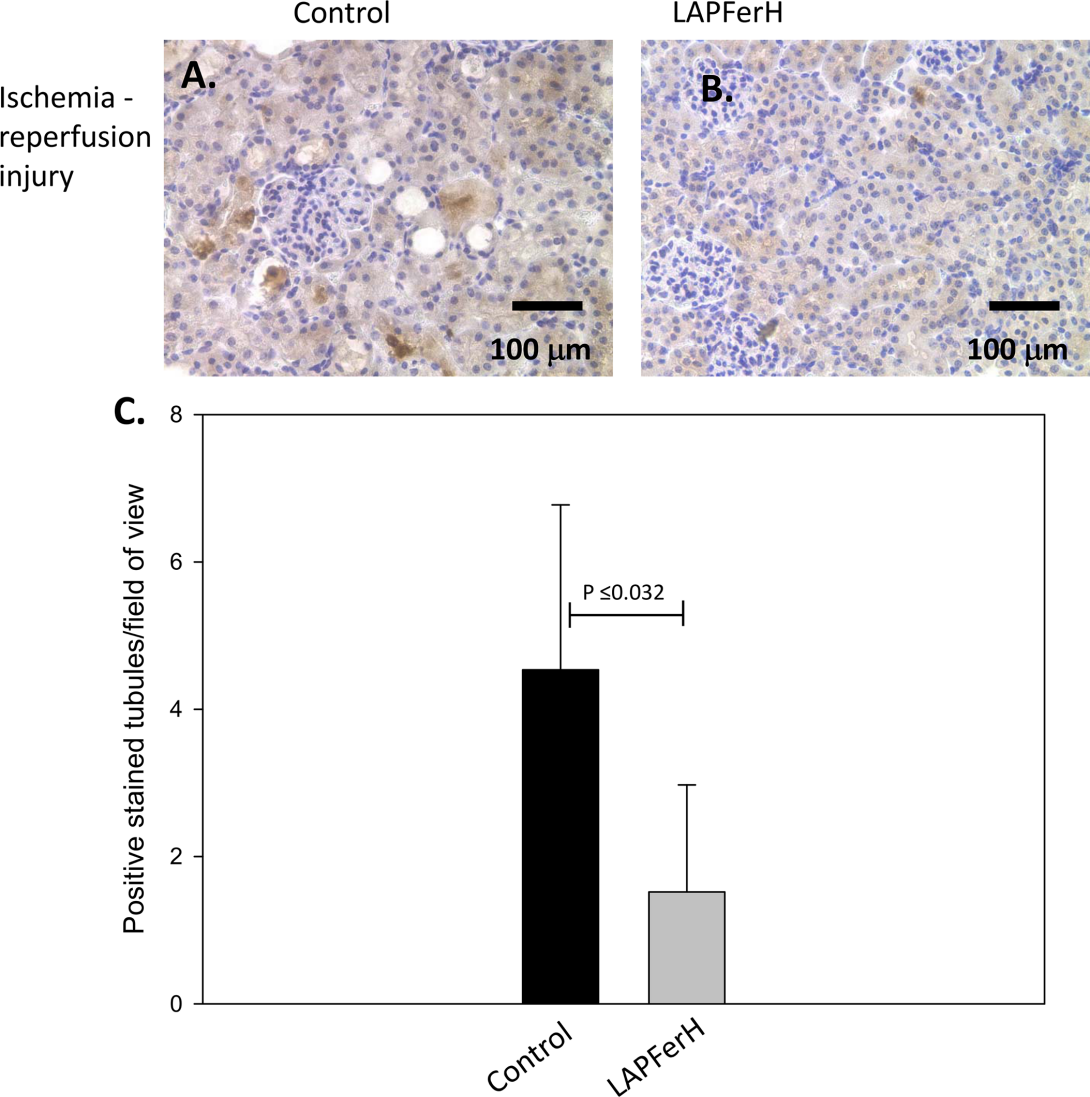
Effect of ferritin H overexpression on apoptosis. Mice were subjected to renal ischemia-reperfusion injury. Representative images of cleaved caspase-3-positive tubules of the cortical kidney of (A.) Control and (B.) LAPFerH mice are shown. Original magnification = x 200. Graph (C.) shows the average number of positively stained tubules per field of view is higher in the Control mice compared to the LAPFerH following ischemia-reperfusion injury (P ≤ 0.0032).

We also measured the effect of ferritin H overexpression on apoptosis by immunohistochemical staining for cleaved caspase-3, a main effector caspase of the apoptotic cascade. Cleaved caspase-3 was detected in 9 of 27 (33.3%) fields of view at x200 magnification of the renal cortex of ischemia-reperfused kidney sections of LAPFerH mice (n = 9, representative image shown in Fig 3B) compared to 21 of 30 (70.0%) the control mice (n = 10, representative image shown in Fig 3A). As quantified in Fig 3C, there was a mean of 1.52 ± 1.5 positively stained cells/LAPFerH ischemia-reperfused kidney section vs. a mean of 4.53 ± 2.2 positively stained cells/control ischemia-reperfused kidney section (P ≤ 0.0032).

**Fig 4:**
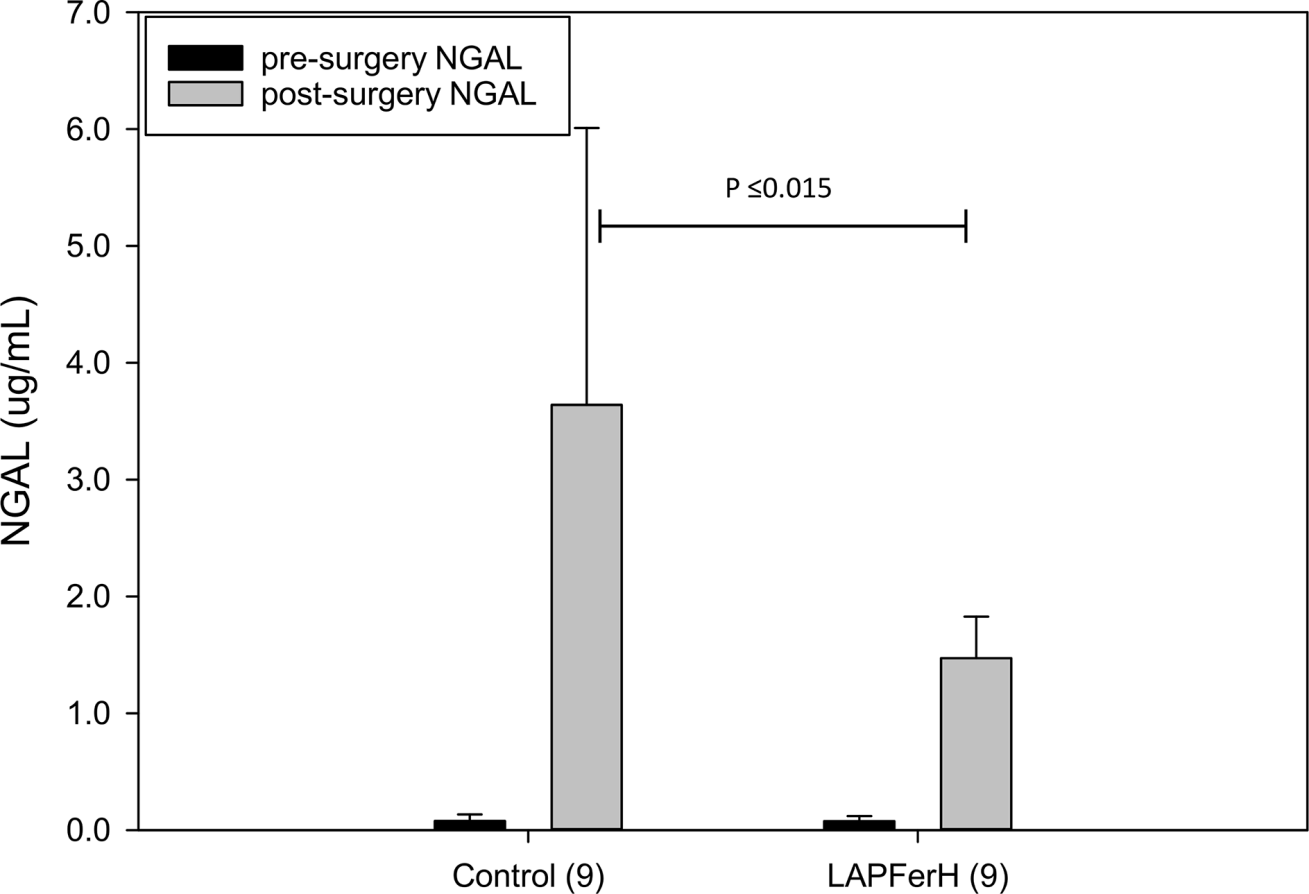
Effect of ferritin H overexpression on urinary NGAL in mice subjected to ischemia-reperfusion injury. Mice were subjected to 45 min ischemia to the right kidney, and then allowed to recover for 24 hr. Urinary NGAL levels were measured prior to ischemia and after 24 hr reperfusion in control mice (n = 9) and ferritin H overexpressing (LAPFerH) mice (n = 9). Data are shown as means ± SD. Urinary NGAL following 24 hr-reperfusion was significantly higher in the Control mice compared to the LAPFerH mice (P ≤ 0.015).

Another measure of IRI-induced renal dysfunction is urinary NGAL [47]. We measured urinary NGAL levels preoperatively and 24 hr postoperatively by ELISA (Fig 4). Preoperatively, mean urinary NGAL level for all animals studied was 0.075 ± 0.049 ug/mL. Preoperative urinary NGAL level was not different in LAPFerH mice (0.075 ± 0.044 ug/mL, n = 9) compared with the control mice (0.076 ± 0.056 ug/mL, n = 9). Although both treatment groups showed significant elevation of urinary NGAL levels following 24-hours reperfusion compared to the preoperative urinary NGAL levels, urinary NGAL was significantly lower among the LAPFerH mice compared to the control group (1.47 ± 0.36 ug/mL versus 3.64 ± 2.37 ug/mL, respectively, P ≤ 0.015).

### Ferritin H mitigates the oxidative stress response

**Fig 5:**
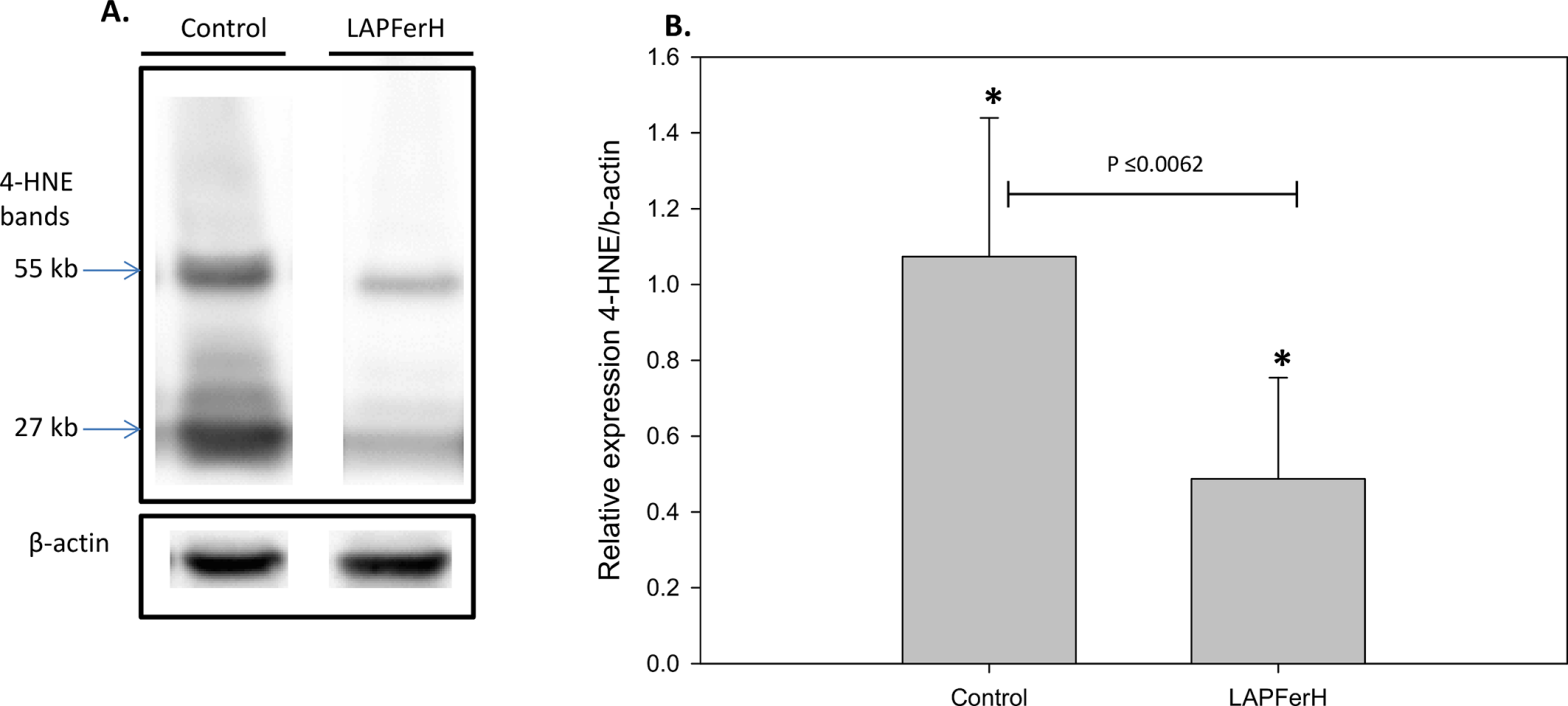
Expression of 4-HNE-protein adducts is reduced in ischemia-reperfused kidney lysates in ferritin H overexpressing (LAPFerH) mice. (A.)Representative immunoreactive bands are shown for Control and LAPFerH kidney protein lysates. (B.) Densitometry of relative expression of 4-HNE adducts in the ferritin H overexpressing mice (LAPFerH; n = 6) compared to the Control mice (n = 8)(*P≤0.0062).

Although the measurements described above demonstrate substantial reduction of renal injury in ferritin H overexpressing mice, they do not provide a mechanism for the renoprotective effect of ferritin H. To directly test whether ferritin H overexpression achieved its cytoprotective effect through amelioration of oxidative stress, we examined 4-HNE-protein adducts, a marker of oxidant stress in the kidney. Reinstitution of oxygen to the ischemic kidney initiates various processes leading to generation of reactive oxygen species (ROS), including nitric oxide (NO) and superoxide (O2−) [48], which react to form the powerful nitrating agent peroxynitrite that leads to a host of injurious events, including lipid peroxidation and the formation of 4-hydroxy 2-nonenal (4-HNE)-protein adducts [49–51]. Immunoblot analysis was used to monitor 4-HNE-protein adducts in ischemia-reperfused kidneys (Fig 5A). Several immunoreactive bands were visualized in both LAPFerH and control kidney lysates; however, the intensity of staining was significantly reduced in the ferritin H overexpressing mice compare to the control mice (P<0.0062)(Fig 5B).

## Discussion

In this study, we investigated the role of ferritin H overexpression in an *in vivo* model of ischemic acute renal failure. We demonstrate that induction of ferritin H in the kidney attenuates histopathological evidence of renal injury, reduces markers of oxidative stress, and attenuates the decline in renal function observed with ischemia reperfusion.

Ferritin is a critical part of a tightly orchestrated cellular response to oxidative stress. Under conditions of oxidative stress, ferritin is induced at both the mRNA and protein level [52], reducing production of ROS and inhibiting apoptosis [52–55]. Thus, ferritin participates in a feedback loop that limits the degree of damage to an oxidant insult.

Since renal ischemia reperfusion injury is ROS dependent, we hypothesized that increased cellular level of ferritin H prior to ischemia would reduce ROS and attenuate renal injury. Using a double transgenic mouse model [36] where ferritin has been substantially induced prior to the oxidative insult caused by renal ischemia reperfusion injury, we evaluated the ability of H-rich ferritin to have a preemptive impact on kidney damage. Our functional and histological assessments indicate that ferritin H preserves renal function and promotes tubular viability. Further, we demonstrate that the presence of 4-HNE-protein adducts, the major toxic product of lipid peroxidation, was markedly reduced in the ischemia-reperfused kidney tissue of the ferritin H overexpressing mice compared to the control animals. The combination of the number of intact nuclei and luminal casts in the tubules of the kidney tubulointerstitial region and expression level of 4-HNE provides quantitative and qualitative evidence of the amelioration of oxidative stress damage.

The overproduction of ROS in the renal tubular cells caused by ischemia reperfusion injury may also be responsible for the apoptotic death of these cells [56, 57]. Caspase-3 is an important enzyme involved in inflammatory and apoptotic events associated with renal ischemia reperfusion injury [58]. We demonstrated that ischemia reperfusion injury increased cleaved caspase-3 in tubulointerstitial areas. Cleaved caspase-3 was significantly reduced in the kidneys of the ferritin H overexpressing mice compared to the control animals. Our results are consistent with an elegant study demonstrating increased cleaved caspase-3 expression in proximal tubule–specific ferritin H-knockout mice following cisplatin-induced injury [22].

Urinary neutrophil gelatinous-associated lipocalin (NGAL) is an endogenous antimicrobial protein [59]. The canonical role of NGAL is to sequester bacterially-derived iron-binding siderophores, such as enterochelin [60]. NGAL is present at low amounts in the serum and urine (ca 20 ng/mL); levels in the urine can increase 15-25-fold in response to kidney injury [61]. Although the mechanism of increase is incompletely understood, NGAL is expressed in damaged nephrons in response to stimuli such as depletion of ATP, exposure to hydrogen peroxide, or exposure to bacteria [59]. NGAL is increased within hours after ischemia and has become a validated end-point in mouse models of renal injury, nephrotoxicity, and other nephropathies [40, 62]. We observed that ischemia-reperfused kidneys from control mice had a greater impairment of renal function as evidenced by urinary NGAL levels that were significantly higher than those mice with ferritin-H overexpression (LAPFerH mice). Since NGAL is not known to be regulated by the iron storage capacity of the nephron, we interpret these results to indicate a cytoprotective role of ferritin H in reducing kidney injury.

Iron and oxidative stress have been implicated in a number of common ailments, including diabetes, cardiovascular disease, atherosclerosis, neurodegenerative diseases and cancer[63]. Elucidating the contribution of ferritin to limiting renal ischemia reperfusion injury and other pathologies involving oxidative stress may allow us to precisely define and perhaps ultimately control the expression/delivery of ferritin for therapeutic benefit. Our model system provided the unique ability to test whether induction of H-rich ferritins prior to renal injury could attenuate the degree of injury. This approach has important clinical implications. Hospitalized patients at high risk for renal injury can, at least in part, be prospectively identified based on their underlying renal status, the nature of the surgical procedure, etc.[47]. Further, ferritin H levels can be modulated *in vivo* in experimental animals; for example, ferritin levels in the liver are increased by cancer chemopreventive dithiolethiones [64, 65]. Thus, further study focused on increasing ferritin expression in patients at high risk for renal injury may provide insight into the clinical benefit of ferritin in ameliorating renal damage from ischemia reperfusion.
